# New Appliances and Things Medical

**Published:** 1904-12-17

**Authors:** 


### NEW APPLIANCES AND THINGS MEDICAL.

[We shall be glad to receive at our Office, 28 & 29 Southampton Street, Strand, London, W.C., from the manufacturers, specimens of all new preparations and appliances which may be brought out from time to time.]

### PHILLIPS'S MILK OF MAGNESIA.

(C. H. Phillips Chemical Co., 14 Henrietta Street, Covent Garden, London, W.C.)

As a means of obtaining the antacid action of magnesia this is a very useful and successful preparation. It is a pure hydrate of magnesium, and thus does not, like the carbonate, liberate carbonic acid gas in the stomach and cause a sense of distension. Containing 24 grains of the hydrate to the ounce, it is much stronger than the ordinary fluid magnesias.

### SPARKLETS.

(Sole Makers: Aerators, Limited, Angel Road, Edmonton, London, N.)

The use of Sparklets for producing aerated waters of various kinds is becoming decidedly more popular, and deservedly so, because it is always possible to obtain an analysis of the water used in any household, and so the consumer may easily assure himself that only pure water is used in the manufacture of these aerated waters; and also that they have been freshly prepared. It has occasionally, but we should imagine very rarely, happened that the sparklet has caused a slightly ferruginous taste or appearance to be communicated to the water, and this may have militated against their use to some extent. The cause of this was a trace, a mere trace is sufficient, of the oxide of iron which the water had derived from the inside of the sparklet case; and most probably it was present in those syphons in which the contents had been allowed to stand too long before being used. At all events the slight, and withal very harmless, drawback of the presence of a little iron has now been corrected by the makers. The improvement consists of a small valve ingeniously inserted in the piercing pin which isolates the steel bulb containing the sparklet and so effectually prevents the solution of that minute quantity of iron which, in the older form, sometimes produced the unpleasant taste complained of. Certainly it was not present in the contents of a syphon we allowed to stand for many days. The shape, size, price and method of use are in no way affected by the new contrivance, and it is worthy of remembrance that the newer patterns of syphons are made to gauge and have all their parts interchangeable.

### MALTINE WITH CREASOTE.

(Maltine Manufacturing Co., Ltd., 24 Hart Street, Bloomsbury, London, W.C.)

The excellence of maltine as a nutritive and digestive agent is well known, and it has already secured recognition as a suitable vehicle for various medicines. In the present instance it is combined with creasote, and the result, from a pharmaceutical standpoint, is everything that can be desired. The same verdict, too, may be pronounced in reference to the medicinal value of the preparation, and though creasote cannot be regarded as other than a nauseous medicine, many patients will be found to take it without serious objection when combined with maltine. The thorough diffusion of the creasote through the maltine basis favours absorption and assimilation, and thus the full medicinal action of the remedy is secured.

